# L19-IL2 Immunocytokine in Combination with the Anti-Syndecan-1 46F2SIP Antibody Format: A New Targeted Treatment Approach in an Ovarian Carcinoma Model

**DOI:** 10.3390/cancers11091232

**Published:** 2019-08-23

**Authors:** Paola Orecchia, Enrica Balza, Gabriella Pietra, Romana Conte, Nicolò Bizzarri, Simone Ferrero, Maria Cristina Mingari, Barbara Carnemolla

**Affiliations:** 1 UOC Immunology Unit IRCCS Ospedale Policlinico San Martino, 16132 Genoa, Italy; 2 UOC Cell Biology, IRCCS Ospedale Policlinico San Martino, 16132 Genoa, Italy; 3 Department of Experimental Medicine (DIMES), University of Genoa, 16132 Genoa, Italy; 4 Molecular Diagnostic Unit, IRCCS Ospedale Policlinico San Martino, 16132 Genoa, Italy; 5 Division of Gynecologic Oncology, Fondazione Policlinico Universitario Agostino Gemelli, IRCCS, Catholic University of Sacred Heart, 00168 Rome, Italy; 6 Academic Unit of Obstetrics and Gynecology, IRCCS Ospedale Policlinico San Martino, 16132 Genoa, Italy; 7 Department of Neurosciences, Rehabilitation, Ophthalmology, Genetics, Maternal and Child Health (DiNOGMI), University of Genoa, 16132 Genoa, Italy; 8 Department of Experimental Medicine (DIMES) and Center of Excellence for Biomedical Research, University of Genoa, 16132 Genoa, Italy

**Keywords:** ovarian cancer, syndecan-1 (SDC1), 46F2SIP anti-syndecan-1, L19-IL2 immunocytokine, vascular mimicry, angiogenesis, vessels normalization, targeted therapy

## Abstract

Epithelial ovarian cancer (EOC) is the fifth most common cancer affecting the female population. At present, different targeted treatment approaches may improve currently employed therapies leading either to the delay of tumor recurrence or to disease stabilization. In this study we show that syndecan-1 (SDC1) and tumor angiogenic-associated B-fibronectin isoform (B-FN) are involved in EOC progression and we describe the prominent role of SDC1 in the vasculogenic mimicry (VM) process. We also investigate a possible employment of L19-IL2, an immunocytokine specific for B-FN, and anti-SDC1 46F2SIP (small immuno protein) antibody in combination therapy in a human ovarian carcinoma model. A tumor growth reduction of 78% was obtained in the 46F2SIP/L19-IL2-treated group compared to the control group. We observed that combined treatment was effective in modulation of epithelial-mesenchymal transition (EMT) markers, loss of stemness properties of tumor cells, and in alleviating hypoxia. These effects correlated with reduction of VM structures in tumors from treated mice. Interestingly, the improved pericyte coverage in vascular structures suggested that combined therapy could be efficacious in induction of vessel normalization. These data could pave the way for a possible use of L19-IL2 combined with 46F2SIP antibody as a novel therapeutic strategy in EOC.

## 1. Introduction

Similar to normal organs, tumors need to establish a blood supply to satisfy their demand for oxygen and nutrients, which are prerequisites for tumor growth and progression [1,2,3]. Solid tumors create a vascular network through several known processes, such as angiogenesis, vasculogenesis, vascular co-option, and vasculogenic mimicry (VM) [4,5]. The resulting tumor blood vessels are structurally and functionally abnormal. They are tortuous, inefficient, and immature, with poor pericyte coverage composed of tumor endothelial cells with loose endothelial junctions [6,7]. These characteristics contribute to a pro-tumorigenic and immunosuppressive environment altering the therapy response of tumor cells [1]. Currently in cancer treatment, one strategy is to use antiangiogenic therapies in association with chemotherapy or radiotherapy to augment their efficacy by benefiting from the vascular “normalization” induced by antiangiogenic therapy [6,8,9]. The vascular “normalization” can be defined as the period of time during or after antiangiogenic therapy when the vascular density is low, blood vessels are highly covered by pericytes, the endothelial junctions are partially recovered, the perfusion is increased, and hypoxia is reduced [6]. This normalization could improve the efficiency of currently employed therapies, such as chemotherapy, radiotherapy, and immunotherapy [1,8]. Epithelial ovarian cancer (EOC) is the second most common and the most lethal gynecologic malignancy in the western world [10,11]. Angiogenesis plays a critical role in the pathogenesis of EOC and it is responsible for tumor spread and metastasis [12,13]. Consequently, molecules that play a key role in promoting angiogenesis, such as vascular endothelial growth factor (VEGF), platelet derived growth factor (PDGF), fibroblast growth factor (FGF), and the angiopoietin/Tie2 receptor complex, are considered good targets for anti-tumor therapies [12]. To date, promising results have been obtained from antiangiogenic therapies, such as bevacizumab a humanized monoclonal antibody against VEGF, or VEGF receptors (VEGFR) inhibitors and several polyADP-ribose polymerase inhibitors (PARPi). However, although different targeted treatment approaches and biological drugs could improve therapy and lead to the delay of recurrence or stabilization of the disease, they have not been shown to cure ovarian cancer [10,14]. Some conventional therapies, such as chemotherapy, radiotherapy, and antiangiogenic therapies, may serve as a catalyst for processes involved in tumor progression, such as VM [15,16,17,18]. VM is referred to as a phenomenon utilized by highly aggressive cancer cells to generate vascular-like structures without the actual presence of endothelial cells to provide blood and nutrition to aggressive tumor tissues [15,17]. VM has been detected in numerous malignant tumors, such as EOC. Moreover, it is widely acknowledged that epithelial-mesenchymal transition (EMT), a process correlated with cancer stem cell (CSC) tumor phenotype, is involved in VM formation [17,19,20,21]. VM has been demonstrated as an unfavorable survival factor and a marker of poor prognosis in various cancers, such as ovarian cancer, inflammatory breast cancer, and gastric adenocarcinoma [17,22,23,24]. Syndecan-1 (SDC1, CD138), one of the four members of the syndecan family, is a cell surface heparan (HS) and chondroitin sulphate (CS) proteoglycan that we showed to be involved in VM of melanoma [25]. SDC1 is expressed predominantly in epithelial cells [26], but it is also found in B lymphocytes at specific stages of differentiation [27]. The structural features of the HS chains are responsible for the interaction of SDC1 with a number of soluble factors, including pro-angiogenic factors, such as VEGF and fibroblast growth factor-2 (FGF-2), cell-associated molecules, and extracellular matrix (ECM) components, such as collagens, fibronectin, and tenascin [26,28,29,30]. Furthermore, elevated levels of VEGF and shed SDC1 form matrix-anchored complexes that together activate integrin and VEGF receptors on endothelial cells, thereby stimulating tumor angiogenesis [31]. SDC1 is absent in normal ovarian tissues, but present in epithelial and stromal cells of benign and borderline tumors. Moreover, the expression of shed SDC1 is a poor prognostic factor of overall survival in patients with ovarian cancer [30,32,33,34,35]. A number of findings suggest that SDC1 is involved in the stimulation of cancer stem cells (CSC), thus affecting disease relapse and resistance to therapy [29]. Previously, we reported that targeting SDC1 using the human recombinant antibody scFv (single chain Fragment variable) OC-46F2 was able to inhibit tumor growth in melanoma and human ovarian carcinoma models induced in NOD SCID (Non-Obese Diabetic Severe Combined Immunodeficiency) mice [36]. Moreover, in a human melanoma xenograft model, we observed that the anti-syndecan-1 antibody OC-46F2, administered either as monotherapy or in combination with the immunocytokine L19-IL2, inhibited tumor growth, inducing a dramatic decrease of vascular density and loss of VM structures [25]. L19-IL2 is an immunocytokine composed of an scFv specific for the angiogenesis-associated B-fibronectin (B-FN) isoform and IL2 (interleukin 2) selectively accumulated on tumor neovasculature, and showed a good anti-tumor activity in preclinical models and PhaseI/II clinical trials, both in solid and hematological tumors [37,38,39,40]. In this study, we show that both SDC1 and B-FN are involved in EOC progression and we hypothesize a possible employment of L19-IL2 and of the new anti-syndecan-1 46F2SIP (small immuno protein) antibody format in combined therapy to treat a human ovarian carcinoma model. We observed that combined treatment was effective in modulation of epithelial-mesenchymal transition (EMT) markers, loss of stemness properties of tumor cells, and alleviated hypoxia, which was in correlation with reduction of VM structures in treated tumors. Moreover, we describe a prominent role of SDC1 in VM, since targeting of this proteoglycan was able to inhibit the formation of tubule-like structures by tumor cells, both in vitro and in vivo. Interestingly, an improved pericyte coverage in vascular structures could indicate that combined therapy was efficacious in induction of vessel normalization.

## 2. Results

### 2.1. Syndecan-1 and B-FN in EOC Patients

**Figure 1:**
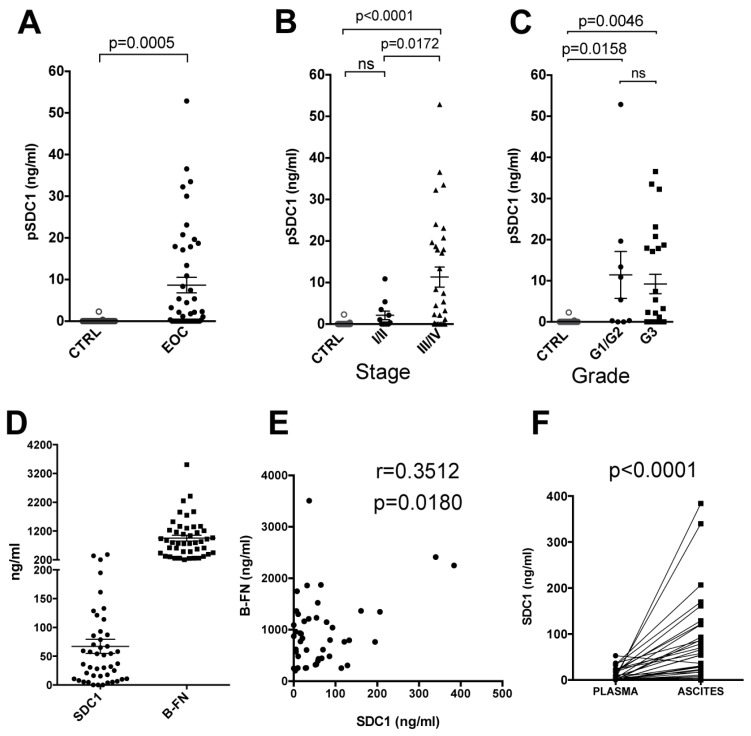
Evaluation of SDC1 (syndecan-1) and B-FN (B-fibronectin) levels in plasma and ascites from EOC patients. (**A**) SDC1 plasma (pSDC1) levels in EOC patients (n = 45) were significantly higher than in healthy donors (n = 29). (**B**,**C**) Distribution of pSDC1 (plasma SDC1) levels at diagnosis in a cohort of 45 EOC patients, stratified according to stage (FIGO, International Federation of Gynecology and Obstetrics) and tumor grade, and in healthy controls. (**D**) SDC1 and B-FN levels in ascites from EOC patients (n = 45). Horizontal bars indicate mean ± SE (Standard error) values for each group. (**E**) A significant correlation (*p* < 0.05) was found between SDC1 and B-FN. Pearson correlation coefficient is shown (r). (**F**) SDC1 levels are higher in ascites than in plasma collected at the same time from a sample of 31 stage III/IV patients (*p* < 0.0001 by paired Student’s *t*-test). P values of statistically significant differences between the groups connected by lines are also reported. Note: ns = no significant differences between the indicated groups.

We evaluated the plasmatic and ascitic levels of syndecan-1 and B-FN by sandwich-type ELISA in a cohort of 69 untreated EOC patients with different pathological characteristics of which the majority belonged to serous histotype (n = 57) (Table 1). As previously reported in patients with solid tumors [41,42], no level of circulating B-FN was detected in collected plasma from EOC patients (data not shown). On the other hand, the mean plasma SDC1 (pSDC1) level in 45 patients, was 8.6 ± 1.8 ng/mL (mean ± SEM), which was significantly higher than in age-matched female healthy donors (n = 29) used as controls, where it is undetectable (*t*-test, *p* = 0.0005) (Figure 1A). Patients were stratified by stage (FIGO, International Federation of Gynecology and Obstetrics classification [43]) and using multiple comparison ANOVA, significantly different pSDC1 levels were found between stage III/IV patients and control group (*p* < 0.0001), and between stage III/IV and stage I/II patients (*p* = 0.0172). No significant differences were observed between stage I/II patients and healthy donors (Figure 1B). When patients were stratified by tumor grade, using the previous statistic test we observed that pSDC1 levels in G1/G2 (*p* = 0.0158) and G3 (*p* = 0.0046) patients were significantly different to the control group. No significant differences were observed between G1/G2 and G3 patients (Figure 1C). At the same time we dosed the presence of shed SDC1 (67.12 ± 12.3 ng/mL) and B-FN (954.2 ± 100 ng/mL) in ascites collected from the same 45 EOC patients (Figure 1D). A positive correlation between SDC1 and B-FN was found when these two proteins were dosed simultaneously in ascites from 45 patients (Pearson’s correlation, r = 0.3512, *p* = 0.0180), indicating an involvement of these two molecules in tumor progression (Figure 1E). To gain information on the possible tumor origin of SDC1, we tested 31 plasma and ascites pairs. Paired t tests showed that SDC1 levels in ascites (81.82 ± 16.5 ng/mL) were significantly higher (*p* < 0.0001) than in plasma (10.37 ± 2.4 ng/mL), suggesting that SDC1 is derived from the original tumor site (Figure 1F). These results confirm that pSDC1 could be a useful marker of ovarian carcinoma and that shed SDC1 and B-FN could be two candidates for target therapy in ovarian carcinoma.

### 2.2. SDC1 and B-FN Involvement in VM Process in Ovarian Carcinoma

**Figure 2:**
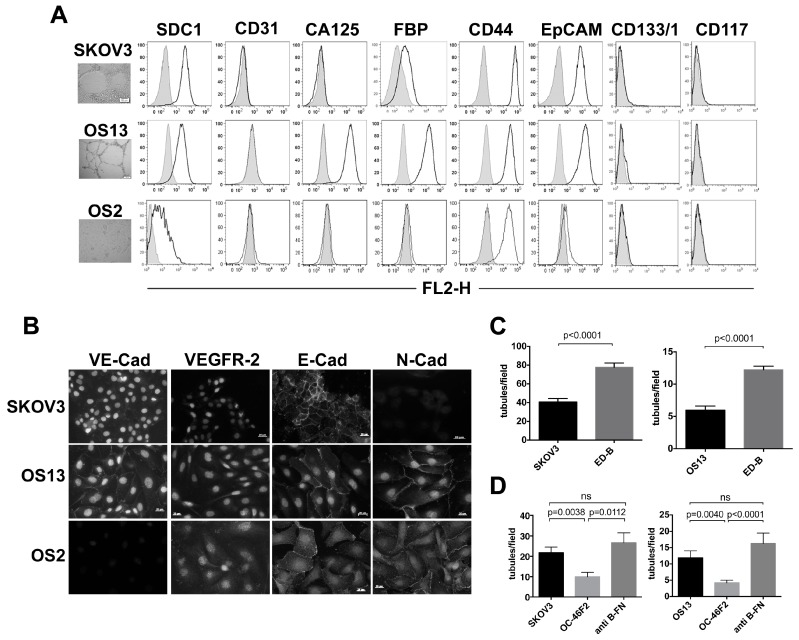
SDC1 and B-FN involvement in the VM process in ovarian cancer. (**A**) Flow cytofluorimetric analysis of SDC1, CD31, CA125, FBP (folate-binding protein), CD44, EpCAM (epithelial cell adhesion molecule), CD133/1, and CD117 expression in VM positive human ovarian carcinoma cell line SKOV3 and serous ovarian carcinoma cell OS13 in comparison to VM-negative clear cell carcinoma cell OS2. Gray profiles represent negative controls. In vitro Matrigel tube formation using SKOV3 ovarian cell line, OS13, and OS2 ovarian cells is reported. Scale bars, 100 μm. (**B**) Immunofluorescence analysis of SKOV3, OS13, and OS2 ovarian carcinoma cells stained with VE-cadherin (VE-cad), VEGFR-2, E-cadherin (E-cad), and N-cadherin (N-cad) counterstained with DAPI (4’,6’-diamidin-2-fenilindolo). Scale bars, 20 μm. (**C**,**D**) In vitro Matrigel tube formation using SKOV3 or OS13 cells in the presence of ED-B or untreated (**C**) and in the presence of scFv OC-46F2 or scFv anti-B-FN in comparison to untreated cells (**D**). The differences in tube formation for different treatments were quantified by column bar graphs. The means ± SE are indicated. Statistically differences *p* values between the groups connected by lines are also reported. Note: ns = no significant differences between the indicated groups.

We isolated adherent tumor cells from 26 ascites of untreated EOC patients that were tested for their ability to form tubule-like structures in Matrigel (VM assay) in comparison to human ovarian carcinoma SKOV3 cell line. In SKOV3 (Figure 2A), 7 out of 26 cell lines classified as mucinous (n = 2) and serous (n = 5) histotype were able to generate tubule-like structures and among these we choose OS13 cells for in vitro experiments. Figure 2A shows cytofluorimetric analysis of SKOV3 cell line and serous ovarian carcinoma OS13 cell line in comparison with clear cell carcinoma OS2 cell line negative for VM assay, as shown by light microscopy photos on the left of the figure. All three cell lines expressed SDC1 and were negative for endothelial cell marker CD31. A strong positivity of ovarian cancer marker CA125 was observed in OS13 only, while ovarian cancer marker folate-binding protein (FBP) was expressed, although at different levels by SKOV3 and OS13. By analyzing the stemness properties of these cell lines, we observed that CD44 was detectable in all cells and EpCAM presented a strong positivity on SKOV3 and OS13 only. In contrast, CD133/1 and CD117 were negative in all the three cell lines. Then, we analyzed ovarian cancer cells for their expression of VM markers VE-cadherin (VE-cad) and vascular endothelial growth factor receptor 2 (VEGFR2) by immunofluorescence. As shown in Figure 2B, VE-cad was expressed in VM-positive SKOV3 and OS13 cells, and not on VM negative OS2 cells. VEGFR2 was expressed by all three cell lines. Moreover, we observed that OS13 and OS2 were characterized by a heterogeneous expression of both E-cadherin (E-cad) and N-cadherin (N-cad), while SKOV3 predominantly expressed the epithelial marker E-cad. We evaluated the levels of SDC1 and B-FN in the culture medium of VM-positive cells SKOV3 and OS13 and of OS2 at 48 h by ELISA test. The results are reported in Figure S1A. The levels of SDC1 increased at 72 h. We cultured ovarian cancer cells SKOV3 and OS13 in the presence of bFGF (basic Fibroblast growth factor) and VEGF for 24 and 48 h. Using cytofluorimetric analysis we observed in OS13 cell line a decrease in percentage of SDC1-positive cells in the presence of b-FGF and VEGF at 24 h (Figure S1B), suggesting an increase of SDC1 shedding. A slightly decrease in percentage of SDC1-positive cells in the presence of ED-B was observed at 24 h. No effects were observed at 48 h. To investigate the role of the ED-B domain of fibronectin in inducing VM, we added recombinant ED-B fragments to ovarian cancer SKOV3 and OS13 cells plated in Matrigel. As shown in Figure 2C, after 24 h ED-B induced a significant increase in tubule-like structure numbers in both cell lines compared to untreated cells. To evaluate the ability of scFv anti-syndecan-1 OC-46F2 and scFv anti-B-FN to inhibit vascular mimicry we performed in vitro experiments in Matrigel using SKOV3 and OS13 human ovarian cancer cells. As shown in Figure 2D, an inhibitory effect of OC-46F2 was obtained by adding the antibody to SKOV3 and OS13 cells that had been cultured for 24 h in Matrigel compared to cells either treated with scFv anti-B-FN or untreated. These results suggest that SDC1 and B-FN are involved in the tubule-like formation of human ovarian cancer cells but only OC-46F2 anti-syndecan-1 antibody was able to inhibit VM.

### 2.3. Expression of Syndecan-1 and B-Fibronectin in Ovarian Carcinoma.

**Figure 3:**
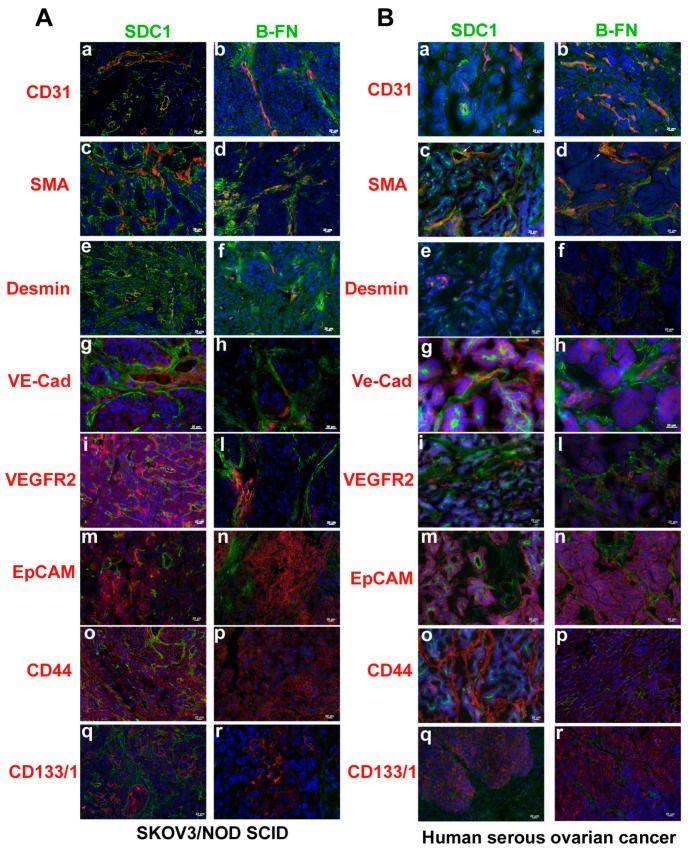
Expression of SDC1 and B-FN in ovarian carcinoma. Immunofluorescence analysis of cryostat sections of SKOV3 induced in NOD SCID mice (**A**) and human serous ovarian carcinoma biopsy (**B**) for SDC1 and B-FN expression with vascular markers CD31, SMA (smooth muscle actin), and Desmin, Vascular Mimicry markers VE-cadherin (VE-cad) and VEGFR2, and ovarian cancer stem cell markers EpCAM, CD44, and CD133/1, counterstained with DAPI. The merged signals are shown. Scale bars, 20 μm.

By double immunofluorescence staining on SKOV3/NOD SCID xenograft ovarian model and human ovarian carcinoma tissues (Figure 3), we analyzed the expression of either SDC1 or B-FN with vascular markers, including CD31, smooth muscle actin (SMA), and Desmin; VM markers, including VEGFR2 and VE-cad; and ovarian cancer stem cell markers, such as EpCAM, CD44, and CD133/1. SDC1 and B-FN were expressed by CD31 positive vessels (Figure 3A,B) at different stages of maturation, as shown by co-localization with SMA (Figure 3A,B) and Desmin (Figure 3A,B), markers of mature and immature vessels, respectively. Moreover, as shown by arrows in Figure 3B, we observed that SMA-positive pericytes were able to express SDC1 and B-FN. SDC1 and B-FN were strongly expressed in vascular structures of human origin identified by anti-human VE-cad (Figure 3A,B). The same distribution was observed for VEGFR2 with SDC1 and B-FN (Figure 3A,B). As shown by double immunofluorescence analysis with anti-EpCAM, anti-CD44, and anti-human CD133/1 antibodies (Figure 3A,B), we observed that SDC1 was accumulated in tumor cells with CSC properties. In contrast, B-FN was localized only in the extracellular matrix around the cancer stem cell niches (Figure 3A,B).

### 2.4. Generation and Characterization of Human 46F2SIP Antibody Format

**Figure 4:**
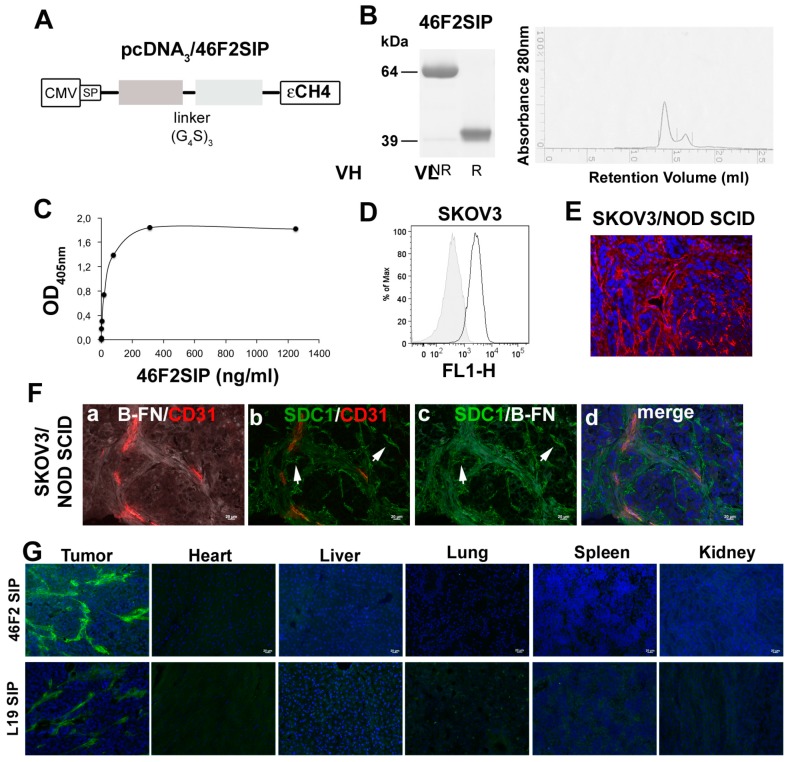
Characterization of 46F2SIP (small immuno protein) antibody format. (**A**) Schematic representation of anti-SDC1 46F2SIP antibody format. (**B**) SDS-PAGE analysis of 46F2SIP in no reduction (NR) and reduction conditions (R) (left panel) and profile of elution of the antibody obtained with FPLC (Fast protein liquid chromatography) indicated that in native conditions the antibody was predominantly present in dimeric form (right panel). The molecular masses (in kilodaltons) of the standards are reported. (**C**) Dose-dependent curve of 46F2SIP to the immobilized human recombinant syndecan-1 extracellular domain. (**D**) FACS (Fluorescence-activated cell sorting) profile of 46F2SIP reactivity on SKOV3 human ovarian carcinoma cells. Gray profiles represent negative controls. (**E**) Immunofluorescence staining of cryostat section of SKOV3 induced in NOD SCID mice using 46F2SIP. (**F**) Immunofluorescence analysis on cryostat sections of SKOV3/NOD SCID for expression of SDC1, B-FN, and CD31. Nuclei were counterstained with DAPI. (**G**) Immunofluorescence of tumor and primary organ samples 12 h after injection of 46F2SIP and L19SIP. Antibodies were detected with anti-IgE (immunoglobulin E) antibody. While in tumors, antibodies were localized in the extracellular matrix and in vessel structures. No accumulation of antibodies was observed in organs. Nuclei were counterstained with DAPI. Scale bars, 20 μm.

Using the gene coding for the variable region of scFv OC-46F2 specific for the extracellular domain of syndecan-1 [36], we prepared the human 46F2SIP antibody in small immuno protein (SIP) format for in vivo application. Figure 4A shows schematic representation of 46F2SIP antibody construct used to express the antibody in mammalian cells. In Figure 4B we show SDS-PAGE analysis (left) and the profile of elution of 46F2SIP obtained with fast protein liquid chromatography (FPLC) (right). Here, 46F2SIP was able to bind to the extracellular domain of syndecan-1 in a dose dependent manner from 1.2 to 1250 ng/mL, reaching a plateau at 1250 ng/mL concentration (Figure 4C). The 46F2SIP antibody maintained the same reactivity of the scFv with SDC1 expressed by human ovarian cancer cell line SKOV3 (Figure 4D) and with tumor cells, neo-angiogenic vascular structures, and ECM in the SKOV3 ovarian carcinoma model (Figure 4E). To evaluate the possible use of the new anti-SDC1 antibody format 46F2SIP in combination therapy with immunocytokine L19-IL2 in human ovarian carcinoma xenograft, we performed a triple immunofluorescence analysis on SKOV3/NOD SCID tumor sections with anti-SDC1 46F2SIP, anti-B-FN scFv that presented the same antigen specificity for B-FN as L19-IL2, and anti-mouse CD31 antibodies. As shown in Figure 4F, anti-B-FN antibody reacted with tumor ECM and neo-angiogenic vessels, while 46F2SIP was able to recognize some tubule-like-structures negative for both mouse CD31 and B-FN (Figure 4F; white arrows). To confirm the distribution in SKOV3 tumor tissues of 46F2SIP and L19SIP, used as control antibody in therapy experiments, tumors and organs (heart, liver, lung, spleen, kidney) were collected at 12 h post antibody injection in NOD SCID mice. Figure 4G shows that 46F2SIP and L19SIP were able to accumulate specifically in tumor stroma and vascular structures, while no accumulation was observed in organs. These observations, together with the fact that the SIP format seems to offer the best compromise in terms of molecular stability, clearance rate, tumor accumulation [44], and the possibility to reduce the amount of administration of 46F2 SIP, induced us to use the 46F2SIP format in therapy.

### 2.5. Therapeutic Efficacy of 46F2SIP in Combination With L19-IL2 Against Human Ovarian Carcinoma Xenograft

**Figure 5:**
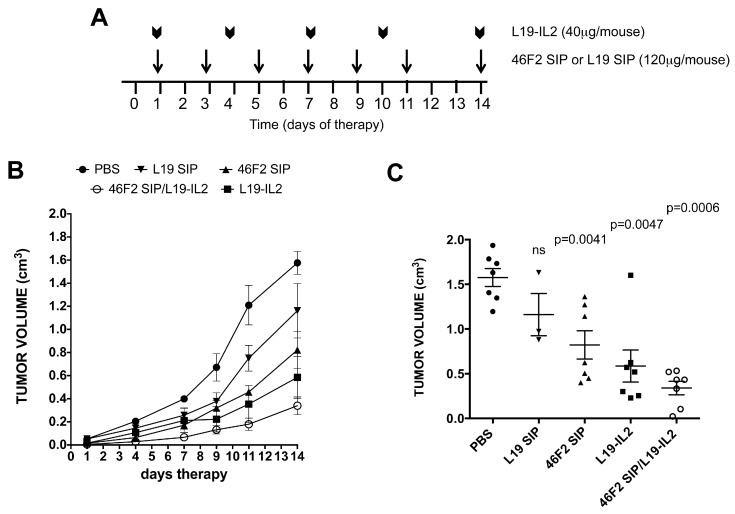
Therapeutic efficacy of 46F2SIP in combination therapy with L19-IL2 in a SKOV3 ovarian carcinoma model. (**A**) Schematic plan for the administration of 46F2SIP or L19SIP and L19-IL2 as monotherapy or in combination therapy. (**B**) Tumor growth inhibition in mice treated with L19SIP, 46F2SIP, and L19-IL2 administered as monotherapy or in combination therapy. (**C**) Comparison of tumor volumes in mice treated with 46F2SIP and L19-IL2 administered as monotherapy or in combination therapy at day 14. Statistically different *p* values between the groups are also reported. Note: ns = no significant differences between the indicated groups. Significance of the differences between the groups was evaluated by nonparametric Mann–Whitney test. The mean tumor volumes ± SE are indicated.

**Figure 6:**
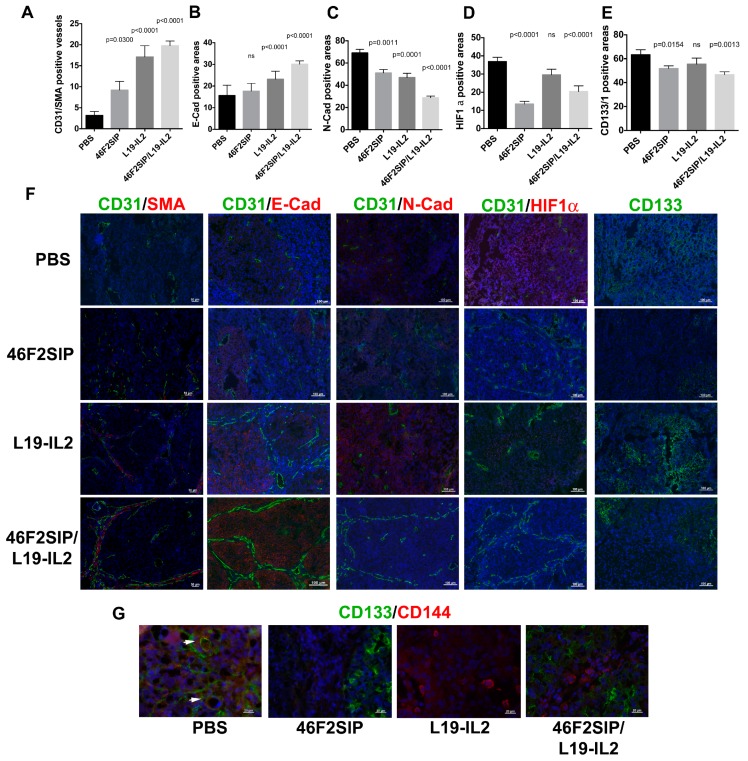
Effects of 46F2SIP/L19IL2 combined therapy on treated tumors. Quantification of CD31/SMA double positive vessels (**A**), E-cadherin (**B**), N-cadherin (**C**) HIF1 alpha (**D**), and CD133/1 (**E**) positive areas from the tumor sections of treated mice, stained as shown (**F**) using ImageJ software. (F) Immunofluorescence analysis of cryostat sections of tumors recovered from SKOV3/NOD SCID mice subjected to the different types of treatment, stained as indicated in each picture and counterstained with DAPI. (**G**) Immunofluorescence analysis of cryostat sections of tumors recovered from SKOV3/NOD SCID mice subjected to the different types of treatments for the expression of cancer stem cell marker CD133/1 and VM marker CD144. Nuclei were counterstained with DAPI. Statistically different *p* values between the groups connected by lines are also reported. Note: ns = no significantly differences between the indicated groups. Scale bars, 20, 50 or 100 m.

We previously reported the therapeutic efficacy of anti-syndecan-1 scFv OC-46F2 on experimental human melanoma and ovarian carcinoma models in NOD SCID mice [36]. Moreover, we observed that in human melanoma xenograft, targeting SDC1 via scFv OC-46F2 enhanced the therapeutic efficacy of L19-IL2, an immunocytokine composed of an scFv specific for the angiogenesis-associated B-fibronectin isoform and IL2, inhibiting tumor growth and VM processes [25]. Combination therapy experiments were performed in NOD SCID mice bearing SKOV3 human ovarian carcinomas. The treatment scheme is reported in Figure 5A and described in Materials and Methods. Figure 5B shows growth curves obtained with anti-syndecan-1 antibody format 46F2SIP used in combination with L19-IL2 immunocytokine or as monotherapy. PBS (phosphate buffer saline) and L19SIP groups were used as controls. It must be noted that tumor development does not have the same growth rates in all mice and that in the same group of animals there is a considerable variability, as we can observe in L19SIP group (Figure 5C). At day 14 of therapy, when the experiment was interrupted to microscopically analyze tumor sections, a 48% tumor growth inhibition rate was obtained in 46F2SIP-treated group versus a 62% of inhibition rate obtained in L19-IL2-treated group, reaching a 78% inhibition rate in 46F2SIP/L19-IL2-treated group. At day 14, compared to untreated group the two-tailed p values of 46F2SIP and L19-IL2 groups were very significant (*p* = 0.0041, *p* = 0.0047) and for 46F2SIP/L19-IL2 group were extremely significant (*p* = 0.0006), indicating the strong efficacy of combined therapy. Moreover, we observed that all seven animals in the combined treatment group presented a tumor volume under 0.6 cm^3^. In particular, in two out of seven animals (28%) tumor volumes were 0.023 and 0.104 cm^3^ (Figure 5C). None of the groups exhibited a body weight loss greater than 3% at any time point during the treatments. All tumors explanted from treated mice with 46F2SIP and L19-IL2 used in combination or as monotherapy were analyzed by immunofluorescence staining in comparison to control group to evaluate the influence of different treatments on pericyte coverage in vascular structures, EMT, and VM processes, and on stemness properties of cancer cells (Figure 6). We did not find differences in vessel density in any treated tumors compared with control group (data not shown). However, all tumors explanted from treated groups exhibited an increase in the number of SMA-positive pericyte coverage vessels compared to control group (Figure 6A). In particular, we observed that in 46F2SIP/L19-IL2-treated group, the CD31/SMA positive vessels revealed normalized morphology, were more linear, less branched, and they were organized to delimitate tumor niches (Figure 6F). The same tumors were analyzed for expression of epithelial markers E-cad and N-cad. Quantification showed a very significant increase of E-cad-positive areas in L19-IL2 and 46F2SIP/L19-IL2-treated tumors compared to the untreated group (Figure 6B), as revealed by the uniform staining pattern of E-cad (Figure 6F). On the contrary, we observed a significant reduction in N-cad-positive areas in all three treated groups (Figure 6C), as shown by immunofluorescence images, where only small areas were positive for N-cadherin (Figure 6F). We quantified HIF1 alpha and CD133/1-positive areas in all groups of animals to analyze tumor hypoxia and CSC population in treated groups of mice (Figure 6D,E). We observed down-regulation in HIF1 alpha expression and stemness marker CD133/1 in 46F2SIP- and 46F2SIP/L19-IL2-treated tumors. L19-IL2 seemed to have no effect on expression of two markers (Figure 6D–F). As CSC phenotype is involved in the VM process, we analyzed tumors from all treated groups of animals by double staining with anti-human CD144 and CD133/1 antibodies (Figure 6G). In treated tumors we observed the presence of isolated CD144-positive cells that were not organized in vascular structures and had lost CSC phenotypes, which differed from the untreated group, in which we found some double positive vascular structures, as indicated in Figure 6G by arrows. These results indicate that combination therapy was effective in down-regulation of the epithelial–mesenchymal transition (EMT) marker, loss of stemness properties of tumor cells, and alleviated hypoxia, which correlated with loss of VM structures in treated tumors. The improved pericyte coverage in vascular structures indicated that combined therapy could be efficacious in induction of vessel normalization.

## 3. Discussion

**Figure 7:**
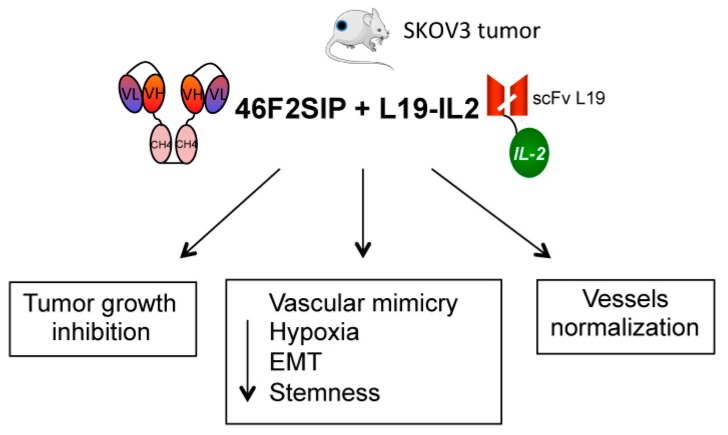
Schematic diagram of 46F2SIP/L19-IL2 combined therapy effects on SKOV3 human ovarian carcinoma model.

EOC is the fifth most common cancer affecting the female population and at present deficiency of diagnostic criteria at early phases along with a lack of effective treatment at advanced stages stand as the most lethal gynecologic malignancies [11]. In previous articles it has been reported that the expression of shed SDC1 was a poor prognostic factor of overall survival in patients with ovarian cancer and a marker for the progression of EOC [33,34,35]. Here, we show that pSDC1 levels were elevated in plasma from EOC patients and completely absent in healthy donors. Moreover, we observed that pSDC1 correlated with tumor aggressiveness and tumor grade. These observations were made in a small cohort and would require further validation on larger numbers of EOC patients. Nonetheless, these data confirm the possible role of SDC1 as a biomarker in EOC. Among new drugs, bevacizumab, an anti-angiogenic compound, and several PARPi were recently approved for ovarian cancer treatment [10]. Vessel normalization induced by anti-angiogenic therapies is now emerging as a novel opportunity to render tumor cells more sensitive to chemotherapy, immunotherapy, and radiation therapy [1,8]. However some conventional therapies may serve as catalysts for processes involved in tumor progression, such as VM, which is one of the reasons for the limited therapeutic effects of existing anticancer drugs [15,17]. Recently we established a possible role of SDC1 in VM of melanoma and that combined therapy could improve the therapeutic efficacy of both anti-SDC1 scFv OC-46F2 antibody and immunocytokine L19-IL2, specific for B-FN, administered as single agents [25,36]. Using the antibody scFv 46F2 to inhibit the tubule-like structures of ovarian cancer VM-associated cells, we established the prominent role of SDC1 in VM, although B-FN was able to induce in vitro VM formation. The biological functions of B-FN are still unclear, however it has been suggested that B-FN increases vascular endothelial growth factor (VEGF) expression, endothelial proliferation, and tube formation [45]. It is noted that tumor cells with CSC characteristics accelerated the VM process [20,21]. We observed that ovarian cancer VM-associated cells were able to secrete SDC1 and B-FN and expressed the stem cell markers CD44 and EpCAM. However, in early in vitro passages, these tumor cells were negative for CD117 and expressed the CD133/1 only when injected in mice, as observed by immunofluorescence analysis and previously described in [46,47]. Furthermore, we observed that these cells were characterized by an epithelial and mesenchymal phenotype simultaneously. It is in accordance with the observations that ovarian carcinomas are heterogeneous tumors containing cells in a hybrid epithelial–mesenchymal state with stem cell-like characteristics modulated in response to the microenvironment [48]. In a comparative study of SDC1 and B-FN expression in ovarian cancer tissues, we observed that some vessels negative for CD31 and anti-B-FN were positive for the anti-syndecan-1 antibody. These preliminary observations and the high SDC1 and B-FN levels dosed in ascite from EOC patients afforded the possibility to investigate the effectiveness of a combined therapy using anti-SDC1 46F2SIP antibody and immunocytokine L19-IL2 in a human ovarian carcinoma model. The SIP antibody format seems to offer the best compromise of molecular stability, clearance rate, and tumor accumulation [44]. Moreover, the possibility to reduce the administration of 46F2SIP in mice induced us to use the SIP format in therapy. The use of human ovarian cell line in immune-compromised NOD SCID mice allowed us to study the human or murine origin of tumor vessels. At day 14 of combined therapy we observed 78% tumor growth inhibition in the 46F2SIP/L19-IL2-treated group of mice. It is known that EMT-inducing transcription factors may induce CSCs to differentiate into tubular-like VE-cadherin^+^ cells in order to form VM channels with ECM remodeling [17,21,22]. The ability of 46F2SIP to down-regulate EMT marker N-cadherin and CSC marker CD133/1 expression, along with loss of human VE-cadherin/CD133 positive vessels in treated mice, could make this antibody a possible candidate for use in EOC target therapy to interfere with the VM process, as observed in vitro. Moreover, owing to the ability of 46F2SIP to alleviate hypoxia, this antibody could overcome the effects of antiangiogenic compounds employed in EOC, such as bevacizumab, which increases HIF1 alpha expression and VM formation [49]. The combined therapy did not affect the number of angiogenic vessels, but induced a significative increase in the number of SMA-positive vessels, suggesting a possible role of therapy in therapeutic normalization that would offer the advantage of creating mature vessels to increase tumor uptake of chemo- and immunotherapy and sensitivity to radiation therapy [9]. A schematic diagram to summarize the effects of 46F2SIP/L19-IL2 combined therapy in SKOV3 human ovarian carcinoma model is reported in Figure 7. Previously, prolonged survival in a patient with recurrent ovarian cancer successfully treated with intra-peritoneal interleukin-2 was reported [50]. When administered locally in the tumor, IL-2 induces the release of pro-inflammatory mediators, increasing sensitivity to further immune attack. This observation strengthens interest in the use of IL-2 in EOC, in particular of IL-2 immunocytokines such as L19-IL2 that should avoid or at least reduce the VLS (vascular leak syndrome) in healthy organs [38,40,51,52]. Moreover, an increased therapeutic potential was reported by combining radiotherapy with L19-IL2 in ED-B-positive tumors [42]. It would be very interesting to evaluate the use of 46F2SIP/L19-IL2 combined therapy in an orthotopic xenograft model of ovarian carcinoma via intraperitoneally injection of ovarian carcinoma cells derived from EOC patients to recapitulate, in part, the patterns of growth and metastasis seen in patients. In accordance with the opinion that simultaneous targeting of multiple pathways involved in tumor progression will be required to treat cancer, we could hypothesize a future therapeutic use of 46F2SIP antibody in combination with immunocytokine L19-IL2 in EOC target therapy, in addition to standard therapeutic protocols [10].

## 4. Materials and Methods

### 4.1. Patients

Clinical samples were obtained upon written informed consent and previous approval by the local ethics committee. All 69 patients showed evidence of previously untreated epithelial ovarian carcinoma (Table 1). Blood samples from 29 tumor-free, age-matched (median = 42 years; range = 27–74) women were used as control. Plasma were collected before surgical procedures and biopsies and ascitic fluids were collected during surgical procedures, respectively. Tumor histopathology, grade, and stage were classified according to the International Federation of Gynecology and Obstetrics (FIGO) criteria.

### 4.2. Cell Lines

Ascites from patients were centrifuged for 10 min at 1700 rpm at room temperature (RT) and supernatants were stored at −80 °C. Cells were suspended in ammonium-chloride-potassium (ACK) lysing buffer for ten minutes in ice and washed two times in RPMI (Roswell Park Memorial Institute) 1640 supplemented with 10% FBS (fetal bovine serum), 2% L-glutamine, and 1% antibiotic mixture (5 mg mL^−1^ penicillin and 5mg ml ^−1^ streptomycin stock solution). Cells were plated in 6-well plates in RPMI 1640 supplemented with 10% FBS, 2% L-glutamine, and 1% antibiotic mixture (5 mg mL^−1^ penicillin and 5 mg mL^1^ streptomycin stock solution). All adherent ovarian carcinoma cells, SKOV3, and Chinese hamster ovary CHO-K1 cell lines were grown at 37 °C in RPMI 1640 supplemented with 10% FBS, 2% L-glutamine, and 1% antibiotic mixture in a 5% CO_2_ incubator. Human ovarian SKOV3 obtained from Biobanking Interlab Cell Line Collection (ICLC), IRCCS Ospedale Policlinico (San Martino, Italy) and human adherent ovarian carcinoma cell OS13 were authenticated by short tandem repeat (STR) profiling.

### 4.3. Flow Cytofluorimetric Analysis

Cytofluorimetric analysis was previously described in [25]. Briefly, 46F2SIP (5 g/mL) mixed with anti-human IgE rabbit antibody (Agilent Technologies, Santa Clara, CA, USA) and the antibodies listed in Supplementary Table S1 were used as primary antibodies. As the secondary antibodies we used PE-conjugated goat anti-mouse IgG1 mAb (monoclonal antibody), anti-mouse IgG2a mAb and anti-mouse IgG2b mAb (Southern Biotechnology Associated, Birmingham, AL, USA), and Alexa Fluor 488 goat anti-rabbit and Alexa Fluor 488 goat anti-rat appropriate as primary antibodies (Thermo Fisher Scientific, Waltham, MA, USA). We cultured ovarian cancer cells SKOV3 and OS13 in the presence of bFGF and VEGF (Thermo Fisher Scientific) at a concentration of 20 ng/mL for 24 and 48 h and analyzed cells by cytofluorimetric analysis for syndecan-1 expression.

### 4.4. In Vitro Tubules Formation Assay

This procedure was previously described in [25]. Human ovarian carcinoma cell SKOV3 and human adherent ovarian carcinoma cells were seeded on Matrigel at a density of 10 ^5^ cells per well in complete specific medium incubated at 37 °C with 5% CO_2_, and were then observed for their capacity to form tubule-like structures. ED-B was added at a concentration of 2 g/mL after two hours when ovarian carcinoma cells started to form tubules, to test the ability of the ED-B fragment of fibronectin to induce tubule formation via SKOV3 and ovarian carcinoma cell OS13. To test the ability of OC-46F2 and anti-B-FN scFv to inhibit tubule formation via SKOV3 and OS13, antibodies were added at a concentration of 200 g/mL when ovarian carcinoma cells started to form tubules. All experiments were performed in triplicate. After fixation in 2% paraformaldehyde in PBS, the tubules formed were counted on ten different high magnification microscopic fields per coverslip under light microscopy (Leica Microsystems, Wetzlar, Germany) at 100X. Images were captured using a DM LB2 microscope camera (Leica) [53].

### 4.5. ELISA Test

For quantitative measurement of human syndecan-1 in ascitic fluids, plasma, and conditioned media of SKOV3, OS13, and OS2, we used the syndecan-1 human ELISA set (ab47352, Abcam, Cambridge, UK) according to the manufacturer’s instructions. To dose B-FN, flat-bottomed 96-well ELISA immunoplates (MaxiSorp, Nunc, Rochester, NY, USA) were incubated overnight at +4 °C with anti-B-FN L19-SIP antibody format [44] at a concentration of 20 g/mL. After 4 washes with phosphate buffer saline (PBS) we dispensed 3% bovine serum albumine in PBS into each well and incubated these at +37 °C for 2 h. After 4 washes with PBS we added the fragment of 120kDa-FN as standard protein (standard curve from 0 ng/mL to 200 ng/mL) or samples to wells in duplicate and incubated these at 37 °C for 2 h. We performed 4 washes with PBS 0.1%Tween-20, then added the monoclonal antibody anti-fibronectin IST4 [54,55] at a concentration of 1.25 g/mL and incubated these at 37 °C for 1 h. After 4 washes with PBS 0,1%Tween-20 we added a horseradish peroxidase-conjugated anti-mouse IgG–IgM antibody (31446, ThermoFisher) at 37 °C for 1 h. After 6 washes with PBS 0,1%Tween-20 distribute ready-to-use TMB (Abcam) we let the reaction develop for 10 min at RT (room temperature). The reaction was stopped with 1M H2SO4 and the absorbance was read at 450 nm with an AD 200 microplate reader (Beckman Coulter, Brea, CA, USA).

### 4.6. Cloning and Expression of 46F2SIP anti-SDC1 and scFv anti-B-FN in Mammalian Cells

In order to construct the 46F2 small immunoprotein (46F2SIP) gene, the DNA sequence coding for the scFvOC-46F2 [36] was amplified by polymerase chain reaction (PCR) using Pwo DNA polymerase (Roche Diagnostics, Milan, Italy), according to the manufacturer’s recommendations, with primers BC-512 (ctcgtgtgcactcggaggtgcagctggtggagtct) and BC-513 (ctctccggagcctaggacggtcagcttggt) containing the ApaLI and BspEI restriction sites, respectively. The amplification product was inserted in ApaLI/BspEI in the pcDNA3.1-epsilonCH4 vector, which provides the scFv gene with a secretion signal required for secretion of proteins in the extracellular medium [44]. This construct was used to transfect Chinese hamster ovary CHO-K1, as previously described [36]. The different geneticin-selected clones were screened by FACS on SKMEL28 human melanoma cell line for their ability to secrete 46F2SIP. The amplification products for scFv CGS1-A1 anti-B-FN [56] and for the genomic sequence of the signal secretion leader peptide [37] were cloned in pcDNA3.1-Myc-His vector and constructs were used for the stable transfection of CHO cell line, as previously reported [36]. The 100% positive clones were cultured in serum-free power-CHO medium (Lonza) and expanded for antibodies and purification.

### 4.7. Protein Purification and Characterization

The 46F2SIP, L19SIP, the immunocytokine L19-IL2m and CGS1-A1 anti-B-FN were purified from the conditioned media of mammalian cells expressing proteins using affinity columns and characterized as previously described [36,37,44,56]. In particular, 46F2SIP was purified on a Protein A/Sepharose column (GE Healthcare) according to the manufacturer’s instructions. The immunocytokine L19-IL2 and L19SIP were purified on the ED-B fibronectin domain conjugated to Sepharose 4B (GE Healthcare). Proteins were dialyzed against phosphate buffer saline (PBS) overnight at +4 °C and sterile filtered using a Millex-GP 0.22 m filter unit (Millipore). Subsequently, they were analyzed under reduction and no reduction conditions by sodium dodecyl sulfate–polyacrylamide gel electrophoresis (SDS-PAGE) and in native conditions by fast-protein liquid chromatography on a Superdex 200 column, as reported in Orecchia 2013 [36]. Monoclonal antibody anti-fibronectin IST4 [54,55] was purified on Protein G/Sepharose 4 Fast Flow antibody purification resin (GeHealthcare, Chicago, USA) according to the manufacturer’s instructions. FN-recombinant fragment ED-B and 120kDa FN were produced and purified as previously reported [56,57,58].

### 4.8. Immunofluorescence

Serial cryostat sections (6 m) from mice tumor samples, immediately snap frozen in OCT (Kaltek, Italy) after removal and from biopsies, were processed for immunofluorescence staining [25]. SKOV3 cells were grown at 37 °C in a 5% CO_2_ incubator, fixed in 2% paraformaldehyde, and permeabilized in 0.1% Triton. For immunofluorescence we used OC-46F2 anti-SDC1 scFv or anti-B-FN scFv (at the concentration of 5 g/mL) mixed with anti-Myc (at a concentration of 1.25 g/mL), 46F2 SIP, or L19-SIP (5 g/mL) mixed with anti-human IgE rabbit antibody or goat anti-human IgE-FITC (Thermo Fisher) the rat anti-mouse CD31 (clone MEC 13.3, kindly provided by A. Mantovani, Humanitas Institute, Milan, Italy), the mouse monoclonal antibody anti-folate binding protein (FBP) (MOV19 kindly provided by S. Ferrini, IRCCS Policlinico San Martino, Genoa, Italy), and primary antibodies, as reported in Supplementary Table S1. We used the Alexa Fluor 488 or 594 goat anti-mouse IgG_1_ for anti-Myc and anti-human CD31, anti-CD133, anti-CD44v6, anti-N-cad, anti-E-cad, and anti-HIF1alpha; the Alexa Fluor 594 or 488 goat anti-rat for anti-mouse CD31; the Alexa Fluor 594 goat anti-mouse IgG_2a_ for anti-SMA; the Alexa Fluor 594 goat anti-rabbit for anti-VEGF receptor-2 and Desmin; the Alexa Fluor 594 goat anti-mouse IgG_2b_ for anti-human CD144 and anti-EpCAM; the Alexa Fluor 488 rabbit anti-goat for anti-human IgE FITC; and the Alexa Fluor 647 goat anti-rabbit for anti-human IgE (ThermoFisher) as secondary antibodies. The tissue sections or cells were counterstained with DAPI using ProLong^®^ Gold Antifade Mountant (Life Technologies) or Glycer gel (DAKO). Images were captured using an ApoTome microscope with AxioCam (Karl Zeiss, Thornwood, NY, USA).

### 4.9. Animal Experimental Models

Female 6-week-old NOD SCID mice (Charles River Laboratories International; Wilmington, MA, USA) were bred and kept under poor conditions at the animal facility of the Policlinico San Martino. All mouse studies were approved by Institutional Animal Care and were cared for in accordance with national legislative provisions for the protection of animals used for scientific purposes. To verify the specific antibody localization in tumor, 10^7^ SKOV3 cells were injected s.c. (subcutaneously) in NOD SCID mice and when tumors were palpable, 280 g 46F2SIP or L19SIP were injected i.p. over two consecutive days. Animals were sacrificed and tumor, spleen, hearth, lung, kidney, and liver were OCT-embedded and stored at −80 °C. In vivo treatments with purified 46F2SIP or immunocytokine L19-IL2 as monotherapy or combination therapy were performed in NOD-SCID mice, each injected s.c. with 10^7^ SKOV3 human ovarian carcinoma cells [59]. When tumors were palpable, different types of therapy were started. For monotherapy and combined therapy, each mice received 120 g 46F2SIP i.p. on alternative days and 40 g L19-IL2 every three days by injection into the tail vein of each animal. Similar groups of animals were untreated or treated with L19SIP used as control. The tumor volumes were determined using the following formula: (d) ^2^ × D × 0.52, where d and D are the short and long dimensions (centimeters) of the tumor, respectively, measured with a caliper. The animals’ weight was recorded daily. Animals were sacrificed when the tumor volume reached a volume between 0.8 and 1.6 cm^3^ and tumors were stored at −80 °C. Specimens of human ovarian carcinoma SKOV3 were obtained by subcutaneous injection (s.c.) of 10^7^ cells/mouse in the hind flank of NOD SCID mice.

### 4.10. Statistical Analysis

Statistical significance of the differences between the groups was evaluated by T-test, ANOVA multiple comparison, or nonparametric Mann–Whitney test using Prism 6 for MAC. All results are presented as mean ± SE.

## 5. Conclusions

We observed that in a human ovarian carcinoma model the combined treatment using L19-IL2 and the new anti syndecan-1 46F2SIP antibody format was effective in modulation of epithelial-mesenchymal transition (EMT) markers, loss of stemness properties of tumor cells and alleviated hypoxia. These effects correlated with reduction of vascular mimicry (VM) structures in treated tumors. These data could pave the way for a possible employ of L19-IL2 combined with 46F2SIP antibody as a novel therapeutic strategy in EOC.

## Figures and Tables

**Table 1:** Distribution of tumor characteristics for EOC (Epithelial ovarian cancer) patients with evidence of disease.

	No. of EOC Cases
**Total**	69
**Age at diagnosis**	
>55	46
<55	14
NA	9
**Stage**	
I	10
II	3
III/IV	46
NA	10
**Histotype**	
SEROUS	57
MUCINOUS	8
CLEAR CELL	4
**Grade**	
1	3
2	12
3	36
NA	18

## Supplementary Materials

The following are available online at https://www.mdpi.com/2072-6694/11/9/1232/s1, Figure S1: (**A**) Levels of SDC1 and B-FN in the conditioned culture medium of SKOV3, OS13 and OS2 at 48 h. (**B**) Surface expression of SDC1 on SKOV3 and OS13 decreases at 24 h in presence of bFGF and VEGF. Percentage of positive cells are reported. List of antibodies used in FCM and IF. Table S1: List of antibodies used in FCM and IF, Table S1: List of antibodies used in FCM (flow cytometry) and IF (immunofluorescence).
