# Supplementary material for: L19-IL2 Immunocytokine in Combination with the Anti-Syndecan-1 46F2SIP Antibody Format: A New Targeted Treatment Approach in an Ovarian Carcinoma Model
Source: Cancers (Basel). 2019 Aug 23;11(9):1232. doi: 10.3390/cancers11091232 (PMC6769537; doi:10.3390/cancers11091232)
Supplement: Supplementary file 1 [file cancers-11-01232-s001.pdf]

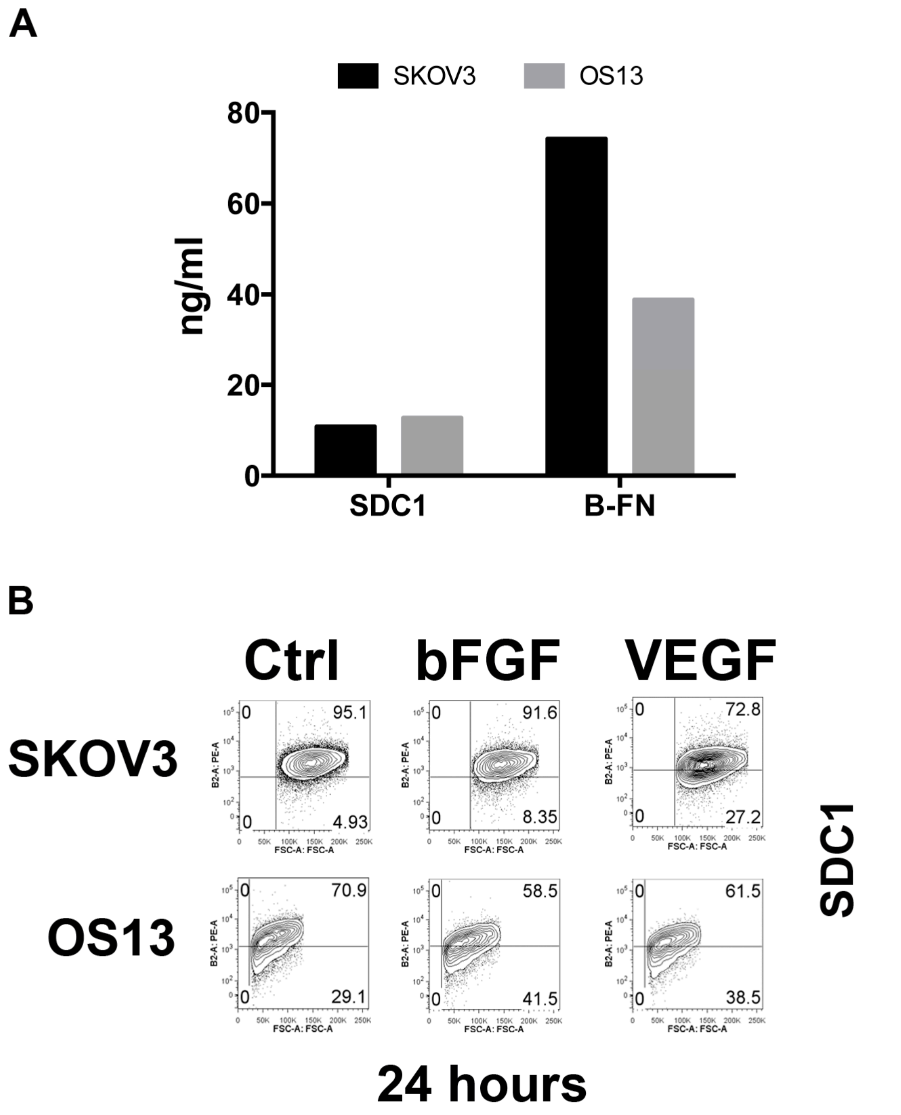

Supplementary Figure S1. (**A**) Levels of SDC1 and B-FN in the conditioned culture medium of SKOV3, OS13 and OS2 at 48 h. (**B**) Surface expression of SDC1 on SKOV3 and OS13 decreases at 24 h in presence of bFGF and VEGF. Percentage of positive cells are reported. List of antibodies used in FCM and IF.

Table S1. List of antibodies used in FCM (flow cytometry) and IF (immunofluorescence).

Specificity	Clone	Manufacturer (location)	Application
human CD31	JC70A	Agilent Technologies (Santa Clara, CA)	FCM
FBP (folate binding protein)	MOV19		FCM
CD44	IM7	BioLegend (San Diego, CA)	FCM
CD133/1	AC133	Miltenyi Biotec (Bergisch Gladbach, Germany)	FCM
CD117 (c-kit)	104D2	BioLegend	FCM
CA125	618F	BioLegend	FCM/IF
EpCAM (epithelial cell adhesion molecule)	9C4	BioLegend	FCM/IF
mouse CD31	MEC 13.3		IF

CD44	MA54	Thermo Fisher Scientific (Waltham, MA)	IF
CD133/1	W6B3C1	Miltenyi Biotec	IF
SMA (smooth muscle actin)	1A4	Agilent Technologies	IF
DESMIN	Polyclonal	Abcam (Cambridge, UK)	IF
CD144	MAB9381	R&D (Minneapolis, MN)	IF
VEGFR2	Polyclonal	Abcam	IF
E-Cadherin	67A4	Santa Cruz biotech. (Dallas, Texas)	IF
N-Cadherin	8C11	Santa Cruz biotech.	IF
HIF1 alpha	ESEE122	Thermo Fisher Scientific	IF
